# Hydrogen-generating Si-based agent protects against skin flap ischemia–reperfusion injury in rats

**DOI:** 10.1038/s41598-022-10228-6

**Published:** 2022-04-13

**Authors:** Naoya Otani, Koichi Tomita, Yuki Kobayashi, Kazuya Kuroda, Yoshihisa Koyama, Hikaru Kobayashi, Tateki Kubo

**Affiliations:** 1 Department of Plastic and Reconstructive Surgery, Osaka University Graduate School of Medicine, Osaka, Japan; 2 Institute of Scientific and Industrial Research, Osaka University, Osaka, Japan; 3 Department of Neuroscience and Cell Biology, Osaka University Graduate School of Medicine, Osaka, Japan; 4 Addiction Research Unit, Development of Novel Diagnosis and Treatment Division, Osaka Psychiatric Research Center, Osaka Psychiatric Medical Center, Osaka Prefectural Hospital Organization, Osaka, Japan

**Keywords:** Experimental models of disease, Disease prevention

## Abstract

Hydrogen is effective against ischemia–reperfusion (I/R) injury in skin flaps. However, the difficulty of continuously administering a sufficient amount of hydrogen using conventional methods has been an issue in the clinical application of hydrogen-based therapy. An Si-based agent administered orally was previously shown to continuously generate a large amount of hydrogen in the intestinal environment. In this study, we assessed the effect of the Si-based agent on the inhibition of I/R injury in skin flaps using a rat model. In the I/R groups, the vascular pedicle of the abdominal skin flap was occluded for three hours followed by reperfusion. In the I/R + Si group, the Si-based agent was administered perioperatively. After reperfusion, flap survival rate, blood flow, oxidative stress markers, inflammatory markers/findings, and degree of apoptosis were evaluated. Flap survival rate was significantly higher, and histological inflammation, apoptotic cells, oxidative stress markers, and levels of inflammatory cytokine mRNA and protein expression were significantly lower, in the I/R + Si group compared to the I/R group. The Si-based agent suppressed oxidative stress, apoptosis, and inflammatory reactions resulting from I/R injury, thereby contributing to improvements in skin flap survival.

## Introduction

Free flap transfer is indispensable for the reconstruction of body defects caused by cancer and trauma. During free flap transfer, free flap tissue is inevitably exposed to an ischemic environment. When tissue that has been in an ischemic environment undergoes reperfusion, tissue damage can occur due to oxidative stress, inflammation, and apoptosis^1–3^. Accordingly, the suppression of ischemia–reperfusion (I/R) injury is important for successful free flap transfer, and various studies have aimed to reduce I/R injury with, for example, the use of agents with anti-oxidative and anti-inflammatory effects, as well as surgical procedures such as ischemic preconditioning^1–8^.

Hydrogen can limit the extent of oxidative stress in tissues by selectively reacting with strong oxidants such as hydroxyl radicals^9^. Many recent studies have attempted the medical application of hydrogen^10,11^. For instance, some studies in the field of plastic surgery have demonstrated the inhibitory effect of hydrogen on I/R injury in flap tissue^12–14^. In these studies, hydrogen was administered mainly in the form of hydrogen water or hydrogen gas. However, since the concentration of hydrogen in water is extremely low (saturated concentration: 1.6 ppm), and hydrogen molecules easily diffuse into the air, there is a limit to the amount of hydrogen that can be taken into the body in the form of hydrogen water^15,16^. Inhalation of hydrogen gas is also problematic given the difficulty of continuously inhaling the gas for a long period, and restrictions on institutions which can use hydrogen gas due to safety issues, since highly-concentrated hydrogen gas is explosive^12,16,17^.

We previously developed an Si-based agent which promotes continuous hydrogen generation in the bowels as a simple and efficient way to introduce hydrogen into the body. Oral intake of this agent generates ≥ 400 ml/g hydrogen (equivalent to ≥ 22 L of saturated hydrogen water) in the body for more than 24 h^16,18^. Moreover, hydrogen atoms, which are formed on the surface of the Si-based agent when it reacts with water in the bowels^19^, have much higher reducing power than hydrogen molecules. The usefulness of this agent has been demonstrated in animal models of oxidative stress-related conditions such as renal failure^20^, Parkinson’s disease^16^, and renal I/R injury^21^.

The present study aimed to examine the effect of the Si-based agent on the suppression of I/R injury in free flap transplantation using a rat model.

## Materials and methods

### Experimental animals

All animal procedures were approved by the animal ethics committee of Osaka University (Approval number 02-086-000) and conducted according to the Guidelines for Proper Conduct of Animals Experiments by the Science Council of Japan and ARRIVE guidelines. Twenty-four adult male Sprague–Dawley rats (CLEA Japan, Inc., Tokyo, Japan) aged 10 weeks and weighing 340–370 g were used in this study. Rats were housed under standard conditions (22–25℃, 12:12-h day/night cycle) with free access to food and water before and after surgery.

### Si-based agent-containing diet

AIN-93 M (Oriental Yeast Co., Ltd., Tokyo, Japan) was used as the normal diet. A special diet containing 1.0 wt% Si-based agent in AIN-93 M was made as described previously^16,20–22^. The Si-based agent reacts with water to continuously generate a high amount of hydrogen in the alkaline intestinal environment. The diet also contains pH-adjusting agents, mainly citric acid, which prevent the Si-based agent from reacting with air moisture during feeding.

### Study design and surgical procedure

Rats were randomly divided into three groups: (1) Sham group (*n* = 8), (2) I/R group (*n* = 8), and (3) I/R + Si group (*n* = 8). Rats in the Sham group and I/R group were fed a normal diet during the experiment. Rats in the I/R + Si group were fed an Si-based agent-containing diet from one week before surgery to the end of the study. Rats in each group were fed in separate cages.

**Figure 1:**
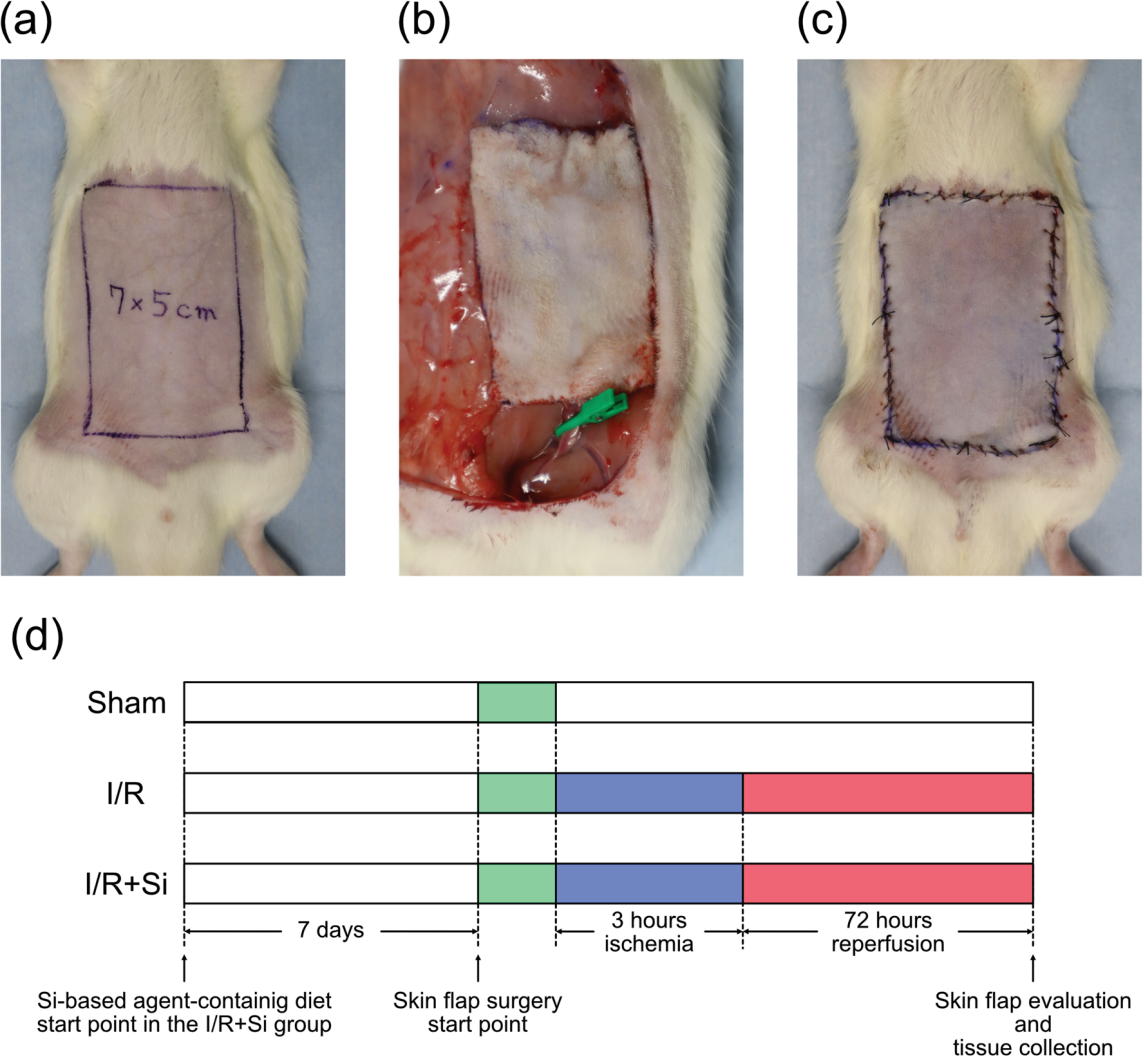
Surgical procedure. (**a**) A 7 × 5 cm rectangular skin flap was designed on the abdomen of the rat. (**b**) The vascular pedicle of the flap was occluded for three hours in the I/R and I/R + Si groups. (**c**) The skin flap was re-sutured in the original position after a silicon sheet was placed between the flap and recipient bed. (**d**) Schematic illustration of the experimental protocol.

The surgical procedure was based on previously reported methods^4,12–14,23^. Briefly, rats were anesthetized by inhalation of isoflurane. A 7 × 5 cm rectangular flap was designed on the abdomen (Fig. 1a). The flap was then elevated along the marked line, and right superficial epigastric artery and vein were ligated so that blood was supplied only by the left pedicle. In the I/R and I/R + Si groups, a microvascular clamp was used to occlude the left superficial epigastric artery and vein for three hours, inducing skin flap ischemia (Fig. 1b). After removing the microvascular clamp and confirming the return of pulsation to the vascular arcade, the skin flap was re-sutured in the original position (Fig. 1c). In the Sham group, the skin flap was not subjected to ischemia: it was re-sutured in the original position immediately after elevation. In all groups, a silicone sheet 0.1 mm in thickness was placed between the flap and the recipient bed before re-suturing to prevent revascularization. The experimental protocol is summarized in Fig. 1d.

### Evaluations of skin flap survival and blood flow

Evaluations of skin flap survival and blood flow after I/R injury were based on previously reported methods^13,14,23^. Seventy-two hours after reperfusion, each rat was anesthetized and fastened to the operation table to expose the entire flap. Skin flap survival rates and blood flow were evaluated by the general observation of survival and necrotic phenomena and a laser speckle blood flow imaging system (Omegazone OZ-1; Omegawave, Inc., Tokyo, Japan). The skin flap survival rate was defined as the ratio of the surviving area to the original flap area. Blood flow was automatically obtained by delineating the surviving area in the image using OZ-1, expressed in PU (mL/min/100 g). After evaluation of skin flap survival rates and blood flow, rats were euthanized. Tissue was obtained from the proximal area of the vascular axis of the flap for further analysis.

### Histological analysis

Tissue samples were fixed with 4% paraformaldehyde, embedded in paraffin, and cut into 4 µm sections. Sections were deparaffinized and subjected to hematoxylin & eosin (HE) and terminal deoxynucleotidyl transferase-mediated deoxyuridine triphosphate nick-end labeling (TUNEL) staining. Histologic changes in HE-stained slides were evaluated using the tissue damage score, as previously described^5,7^, with minor modifications. Specifically, tissue damage was scored according to pathologic findings, including inflammatory cell infiltration, hyperemia, and intra-vascular microthrombosis as follows: 0 (none), 1 (trivial), 2 (mild), 3 (moderate), and 4 (severe). TUNEL staining was performed using the CF®488A TUNEL Assay Apoptosis Detection Kit (Biotium, Inc., Fremont, CA, USA) according to the manufacturer’s instructions. Sections were mounted with VECTASHIELD^®^ Mounting Medium. DAPI (Vector Laboratories, Inc., Burlingame, CA, USA) was used to counterstain cellular nuclei. For quantitative analysis, TUNEL-positive cells in three different slide fields from different skin tissues were counted, as previously described^5,7,13,16,21^. Data are expressed as the percentage of TUNEL-positive cells among the total number of nuclei per field. For histological analysis, a BZ-X800 microscope (Keyence, Osaka, Japan) and Image J (a public domain program developed at the US National Institutes of Health) were used.

### Oxidative stress measurement

The level of malondialdehyde (MDA) in flap tissue was measured as an oxidative stress marker, as previously described^7,8,12^. Briefly, 50 mg of frozen flap tissue was cut into pieces on ice, homogenized in RIPA buffer using a homogenizer, and centrifuged at 10,000 × g for 10 min at 4℃. The supernatant was used for analysis. MDA and total protein measurements were performed using the Amplite™ Colorimetric Malondialdehyde Quantitation kit (AAT Bioquest, Inc., Sunnyvale, CA, USA) and DC™ Protein Assay kit (Bio-Rad Laboratories, Inc., Hercules, CA, USA) according to the manufacturer’s instructions. Optical density was measured using a microplate reader (SH-9000Lab; HITACHI, Tokyo, Japan). MDA level is expressed as nmol/mg protein.

### Real-time quantitative reverse-transcriptase polymerase chain reaction (RT-qPCR) for inflammatory cytokine measurements

RT-qPCR was performed to measure inflammatory cytokine mRNA levels in flap tissue, as previously described^8,24^. Total RNA of flap tissue was extracted using the TRIzol™ Plus RNA Purification Kit (Invitrogen, Carlsbad, CA, USA), according to the manufacturer’s instructions. The concentration of extracted RNA was determined using a spectrophotometer (NanoDrop™ 2000; Thermo Fisher Scientific, Inc., Waltham, MA, USA). Reverse transcription of 1 μg total RNA was performed to synthesize cDNAs using the SuperScript™ IV First-Strand Synthesis System (Invitrogen, Carlsbad, CA, USA), according to the manufacturer’s instructions. RT-qPCR was performed using TaqMan® Gene Expression Assays for IL-1β (Rn00580432_m1), IL-6 (Rn01410330_m1), TNF-α (Rn99999017_m1), and β-actin (Rn00667869_m1), and TaqMan® Gene Expression Master Mix (Applied Biosystems, Waltham, MA, USA) with a QuantStudio™ 7 Flex Real-Time PCR System (Applied Biosystems, Waltham, MA, USA), according to the manufacturer’s instructions. β-actin was used as a control. Expression levels for each gene were evaluated using the 2^−ΔΔCT^ method.

### Inflammatory cytokine measurements by western blotting

Western blotting was performed to measure inflammatory cytokine protein levels in flap tissue, as previously described^24^. Briefly, total protein from the frozen flap tissue was collected and measured. The extracted protein was denatured by heating in sample buffer (EzApply; ATTO Co., Ltd., Tokyo, Japan), loaded onto a 5–20% gradient SDS-PAGE gel (e-PAGEL; ATTO Co., Ltd., Tokyo, Japan), and electrotransferred to a PVDF membrane using iBlot^®^ 2 Dry Blotting System (Invitrogen, Carlsbad, CA, USA). After blocking in 5% nonfat milk in TBST for 30 min, the membrane was incubated with primary antibodies overnight at 4ºC. Rabbit polyclonal anti-IL1β (1:500; bs-0812R, Bioss antibodies, Inc., Woburn, MA, USA), rabbit polyclonal anti-IL6 (1:500; bs-0782R, Bioss antibodies, Inc., Woburn, MA, USA), rabbit polyclonal anti-TNFα (1:500; bs-2081R, Bioss antibodies, Inc., Woburn, MA, USA), and rabbit polyclonal anti-βactin (1:1000; 20536-1-AP, Proteintech Group, Inc., Rosemont, IL, USA) were used as primary antibodies. After washing, the membrane was incubated with HRP-conjugated goat anti-rabbit IgG (1:2000; SA00001-2, Proteintech Group, Inc., Rosemont, IL, USA) for 2 h at room temperature. The bands were visualized using ECL™ Western Blotting Detection Reagents (Cytiva, Marlborough, MA, USA) and ChemiDoc™ Touch Imaging System (Bio-Rad Laboratories, Inc., Hercules, CA, USA). β-actin was used as a control. Relative band densities were measured using Image J.

### Statistical analysis

Tukey’s HSD multiple comparisons test was performed to analyze measurement results using JMP^®^ Pro 16 (SAS Institute Inc., Cary, NC, USA). *P* < 0.05 was considered statistically significant. Data are expressed as mean ± standard error.

## Results

Two rats in the Sham group were excluded from analysis due to partial loss of the surviving flap by self-cannibalism at 72 h after reperfusion. Accordingly, the final analysis was performed with six rats in the Sham group and eight rats each in the I/R and I/R + Si groups.

### Skin flap survival and blood flow

**Figure 2:**
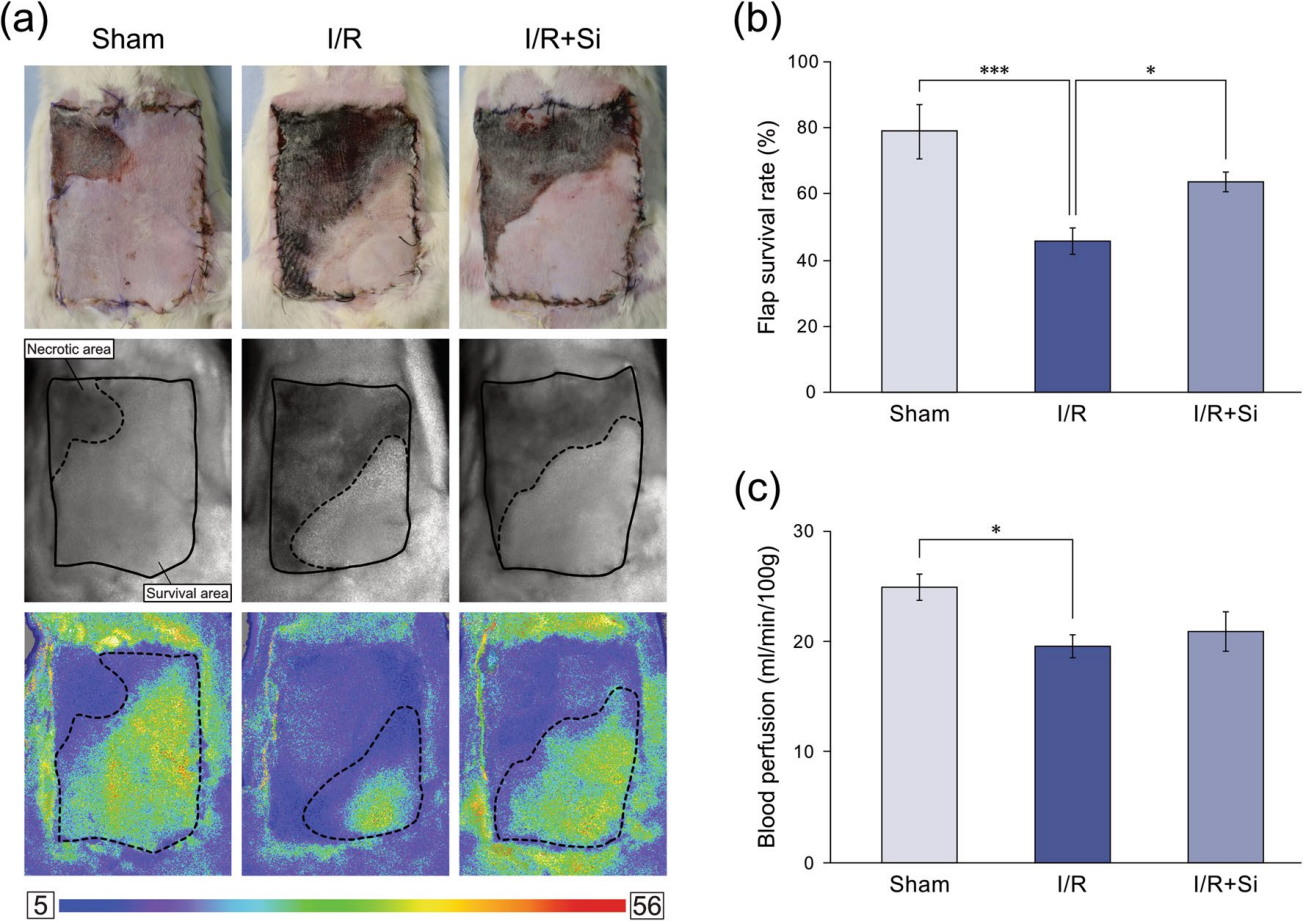
Postoperative skin flap survival and blood flow. (**a**) Representative images of each group, along with blood flow images obtained using the laser speckle blood flow imaging system. Solid lines show the original flap area and dashed lines show the surviving flap areas. (**b**), (**c**) Flap survival rate and blood perfusion after ischemia–reperfusion injury (*p < 0.05, ***p < 0.001; Tukey’s HSD multiple comparisons test, bars represent standard errors of mean).

Seventy-two hours after reperfusion, necrotic flap areas were brown, gray, or black in color and had lost elasticity. In contrast, surviving flap areas were pink or white in color and maintained normal elasticity (Fig. 2a). Mean flap survival rates in the Sham, I/R, and I/R + Si groups were 78.7 ± 8.0%, 45.8 ± 4.0%, and 63.5 ± 3.1%, respectively. The flap survival rate in the I/R group was significantly lower compared to the Sham group (*P* < 0.001). However, the flap survival rate in the I/R + Si group was significantly higher compared to the I/R group (*P* < 0.05), but not significantly different from the Sham group (Fig. 2b). Mean blood flow in the Sham, I/R, and I/R + Si groups were 25.0 ± 1.2 PU, 19.6 ± 1.1 PU, and 20.9 ± 1.8 PU, respectively. Blood flow in the I/R group was significantly lower compared to the Sham group (*P* < 0.05), but not significantly different from the I/R + Si group (Fig. 2c).

### Tissue damage and apoptosis

**Figure 3:**
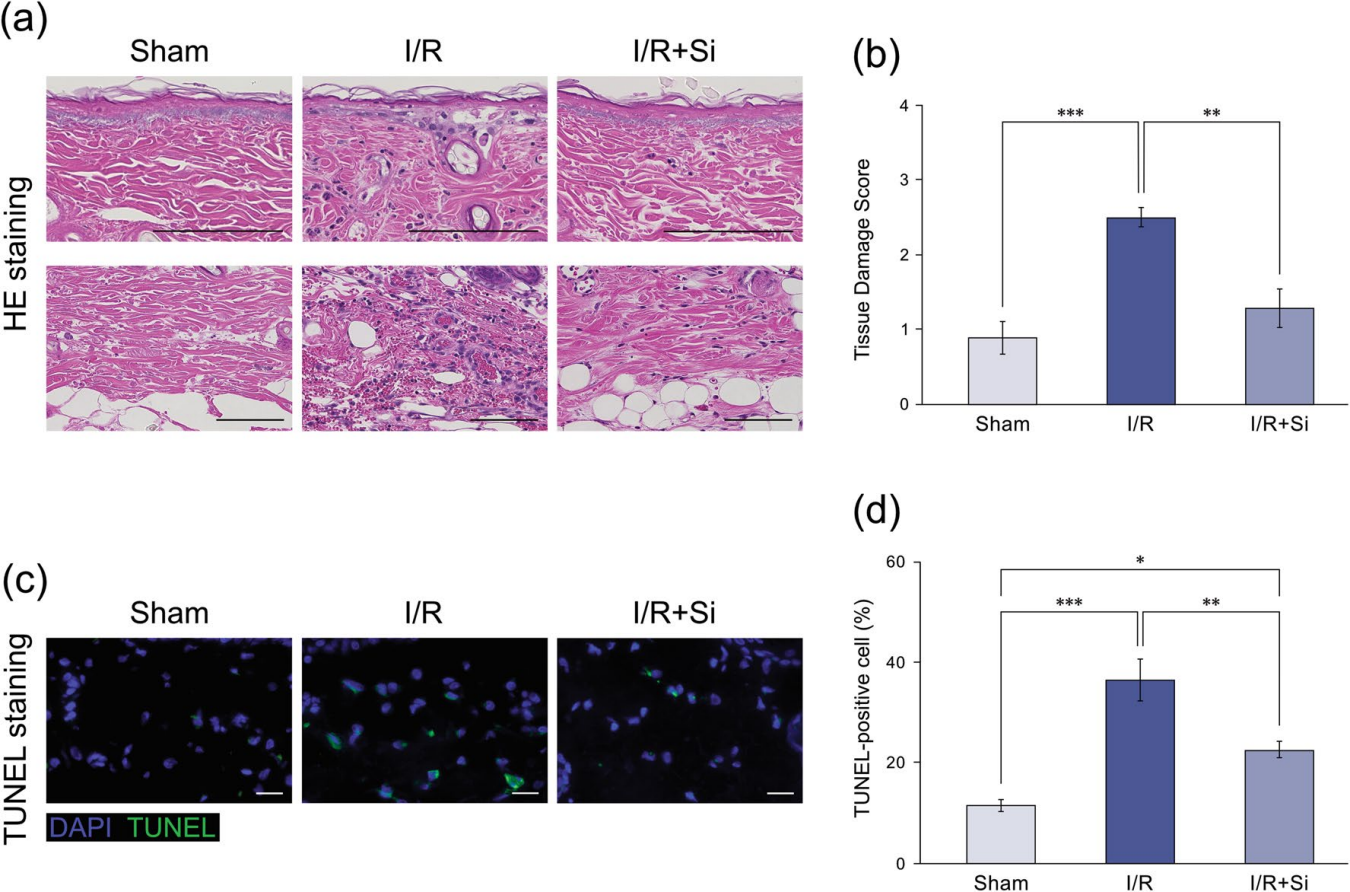
HE and TUNEL staining analysis of skin flap after ischemia–reperfusion injury. (**a**) Representative HE staining images of the superficial and deep layers of the skin flap tissue of each group (scale bar: 100 μm). Inflammatory cell infiltration, hyperemia, and intra-vascular microthrombosis were severe in the I/R group. (**b**) Tissue damage score evaluation. The score for the I/R + Si group was significantly lower compared to the I/R group. (**c**) Representative TUNEL staining images of the dermis of the skin flap tissue of each group (scale bar: 20 μm). (**d**) TUNEL-positive cell evaluation. The percentage of TUNEL-positive cells in the I/R + Si group was significantly lower compared to the I/R group (*p < 0.05, **p < 0.01, ***p < 0.001; Tukey’s HSD multiple comparisons test, bars represent standard errors of mean).

HE staining in the I/R group revealed tissue damage, including inflammatory cell infiltration, hyperemia, and intra-vascular microthrombosis. The tissue damage score in this group was significantly higher compared to the Sham group (*P* < 0.001). In the I/R + Si group, these pathological changes were suppressed and the tissue damage score was significantly lower compared to the I/R group (*P* < 0.01; Fig. 3a,b).

TUNEL-positive apoptotic cells in the Sham, I/R, and I/R + Si groups were 11.5 ± 1.1%, 36.5 ± 4.3%, and 22.6 ± 1.7%, respectively. Proportions of TUNEL-positive apoptotic cells in the I/R and I/R + Si groups were significantly higher compared to the Sham group (*P* < 0.001 and *P* < 0.05, respectively), but was significantly lower in the I/R + Si group compared to the I/R group (*P* < 0.01; Fig. 3c,d).

### Oxidative stress and inflammatory cytokines

**Figure 4:**
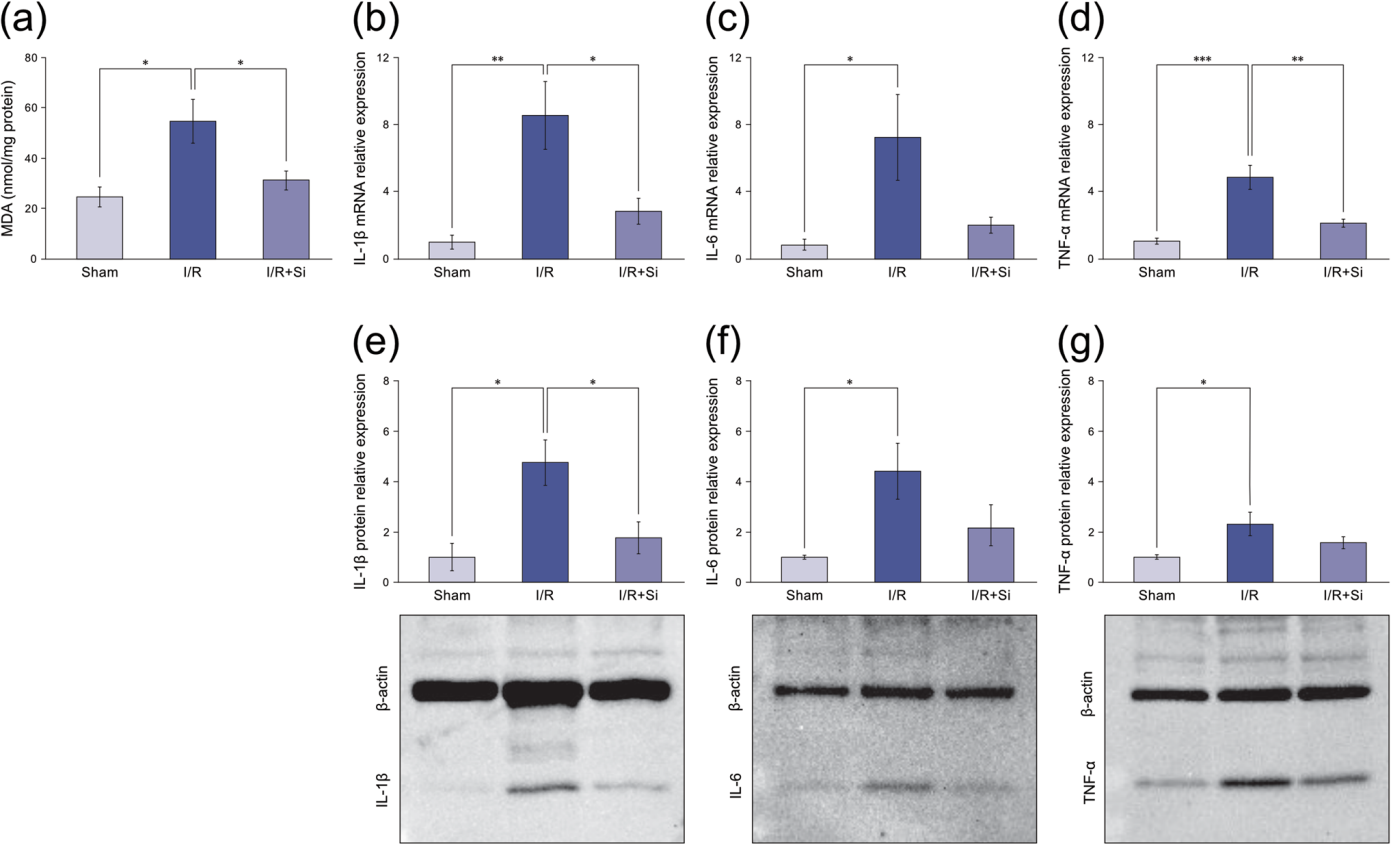
Oxidative stress and inflammatory cytokine measurement after ischemia–reperfusion injury. (*a*) Malondialdehyde (MDA) levels in the flap tissue of the I/R + Si group are significantly lower compared to the I/R group. (**b**)–(**d**) Relative mRNA levels of IL-1β and TNF-α in the flap tissue of the I/R + Si group are significantly lower compared to the I/R group. (**e**)–(**g**) Relative protein levels of IL-1β in the flap tissue of the I/R + Si group are significantly lower compared to the I/R group. Each representative image of Western blotting is presented. Uncropped original images are presented in the Supplementary Fig. 1. All biological replicates are presented in the Supplementary Fig. 2. Full-length blots were absent because the membranes were cut around the predicted molecular weight prior to hybridization with antibodies. (*p < 0.05, **p < 0.01, ***p < 0.001; Tukey’s HSD multiple comparisons test, bars represent standard errors of mean).

Mean MDA levels of flap tissue in the Sham, I/R, and I/R + Si groups were 24.9 ± 4.1 nmol/mg protein, 55.1 ± 8.7 nmol/mg protein, and 31.5 ± 3.7 nmol/mg protein, respectively. MDA levels in the I/R group were significantly higher compared to the Sham group (*P* < 0.05), and levels were significantly lower in the I/R + Si group compared to the I/R group (*P* < 0.05; Fig. 4a). Relative mRNA and protein levels of IL-1β, IL-6, and TNF-α in the I/R group were significantly higher compared to the Sham group. Relative mRNA and protein levels of these cytokines were lower in the I/R + Si group compared to the I/R group. In particular, mRNA and protein levels of IL-1β and mRNA level of TNF-α were significantly lower in the I/R + Si group compared to the I/R group (*P* < 0.05, *P* < 0.05 and *P* < 0.01, respectively; Fig. 4b–g).

## Discussion

The present study demonstrated that oral administration of an Si-based agent suppressed I/R injury in skin flaps in a rat model and increased the survival rate of skin flaps after three hours of ischemia.

Free flap reconstruction is an essential procedure in plastic and reconstructive surgery. With recent improvements in surgical techniques, the success rate of free flap transfer has been reported to be 90–99%^25^. However, I/R injury, which can cause total or partial necrosis of the free flap, is still a challenging problem. Numerous studies have suggested that I/R injury in skin flaps involves oxidative stress, inflammation, and apoptosis. In particular, oxidative stress caused by reactive oxygen species is considered to be the main contributing factor^1–3^. Although the use of various antioxidant, anti-inflammatory, and anti-apoptotic agents^1–3,5,7^, growth hormone^8^, stem cells^6^, and surgical techniques such as ischemic preconditioning^4^ have been reported as means to prevent I/R injury in skin flaps, there is no clear consensus regarding which is optimal. This might be attributed to the side effects associated with the agents, as well as the fact that the surgical techniques require extra operative time and surgical procedures dealing with vascular pedicles can cause vascular endothelial damage, thrombus formation, and vasospasm^2,3,5^.

Hydrogen reduces oxidative stress in tissues by selectively reacting with hydroxyl radicals, which are reactive oxygen species with strong oxidative power. A number of studies have demonstrated the effects of hydrogen on preventing I/R injury in organs such as the brain^9^, heart^26^, liver^27^, intestines^28^, and kidneys^29^. In the field of plastic and reconstructive surgery, hydrogen has been shown to prevent I/R injury in skin flaps^12–14^ and skeletal muscles^30^. Hydrogen is harmless to the human body, and even if ingested in excessive amounts, it is discharged from the body. Thus, the possibility of side effects associated with its administration is low, an advantage compared to other agents^15^. Routes of administration in previous studies assessing the effect of hydrogen on preventing tissue I/R injury were intraperitoneal or intravenous for hydrogen rich saline^12,13,28,29^, or inhalation for hydrogen gas^9,14,24,26,27,30^. However, with these routes, continuous administration of sufficient amounts of hydrogen is difficult. The Si-based agent used in the present study, in contrast, continuously generates a large amount of hydrogen in the intestinal environment, and the agent has been shown to be effective against animal models of various chronic diseases, such as renal failure^20^, Parkinson’s disease^16^, and varicocele-induced abnormal sperm parameters^22^, as well as acute conditions such as renal I/R injury^21^.

In the present study, significantly higher levels of the oxidative stress marker MDA, histological inflammatory cell infiltration and tissue damage, and inflammatory cytokine mRNAs and proteins such as IL-1β, IL-6, and TNFα were observed in flap tissue of the I/R group compared to the Sham group. However, oral administration of the Si-based agent was able to suppress the changes observed in the I/R group, indicating that hydrogen generated from the Si-based agent can prevent the effects of I/R injury in skin flaps, much in the same way they can be prevented by conventional administration of hydrogen water and gas inhalation. Thus, the Si-based agent, which has been shown to be effective against kidney and brain diseases, is also effective against skin and soft tissue diseases.

Oxidative stress and I/R injury are observed in many pathological conditions related to skin and soft tissue. For example, atopic dermatitis and radiation-induced dermatitis are thought to involve oxidative stress. Moreover, I/R injury has been implicated in the worsening of deep tissue injury caused by pressure ulcers, for which hydrogen has been shown to be an effective treatment^24,31–33^. Based on our present results, the Si-based agent may be effective in the prevention and treatment of these conditions as well. In addition to these skin and soft tissue conditions, the Si-based agent may also be effective against I/R injury in other organs for which hydrogen has been shown to be an effective treatment.

This study has some limitations. First, we were unable to examine changes in the treatment effect by ischemic time, or by dosage of the Si-based agent or treatment duration. Second, we could not directly measure the actual amount of hydrogen reaching the assessed tissue. The ischemic time of the skin flap was set to three hours based on previous reports^12,14,23^, but it is unclear whether the reperfusion injury suppression effect of the Si-based agent would be effective for longer ischemic times. The concentration of the Si-based agent in the diet and duration of administration used in the present study were also based on previous reports^21^. One study^20^ reported that the efficacy of the Si-based agent was dose-dependent. Although further studies are needed to determine the optimal concentration of the Si-based agent for each tested condition, the advantage of the Si-based agent is that it is not absorbed by the body and the excess unused hydrogen is discharged, keeping adverse events to a minimum even when used at high concentrations. As for the timing of hydrogen administration for I/R injury, there are two possibilities: preconditioning and postconditioning. While the question of whether preconditioning or postconditioning is more important is a matter of debate^23,30^, the Si-based agent generates hydrogen continuously and is useful in that it can be administered prior to surgery. However, since the Si-based agent generates hydrogen in an alkaline environment in the intestines, there is a time lag for the agent to reach the intestines and begin generating hydrogen. Therefore, it may be difficult to use the agent for unforeseen and emergent conditions such as replantation of amputated limbs.

## Conclusion

The Si-based agent was found to effectively suppress I/R injury in skin flaps, and allow for the production of a large amount of hydrogen continuously via oral administration with a low likelihood of adverse events. Accordingly, the Si-based agent may be suitable for clinical use and has the potential to dramatically expand the scope of hydrogen-based therapy.

## Supplementary Information

Supplementary Information.

## Data Availability

The datasets generated and/or analyzed during the current study are available from the corresponding author on reasonable request.
