# Supplementary material for: Hydrogen-generating Si-based agent protects against skin flap ischemia–reperfusion injury in rats
Source: Sci Rep. 2022 Apr 13;12:6168. doi: 10.1038/s41598-022-10228-6 (PMC9008008; doi:10.1038/s41598-022-10228-6)
Supplement: Supplementary file 1 — Supplementary Information. [file 41598_2022_10228_MOESM1_ESM.pdf]

Hydrogen-generating Si-based agent protects against skin flap ischemia-reperfusion injury in rats

Naoya Otani, Koichi Tomita, Yuki Kobayashi, Kazuya Kuroda, Yoshihisa Koyama, Hikaru Kobayashi, and Tateki Kubo

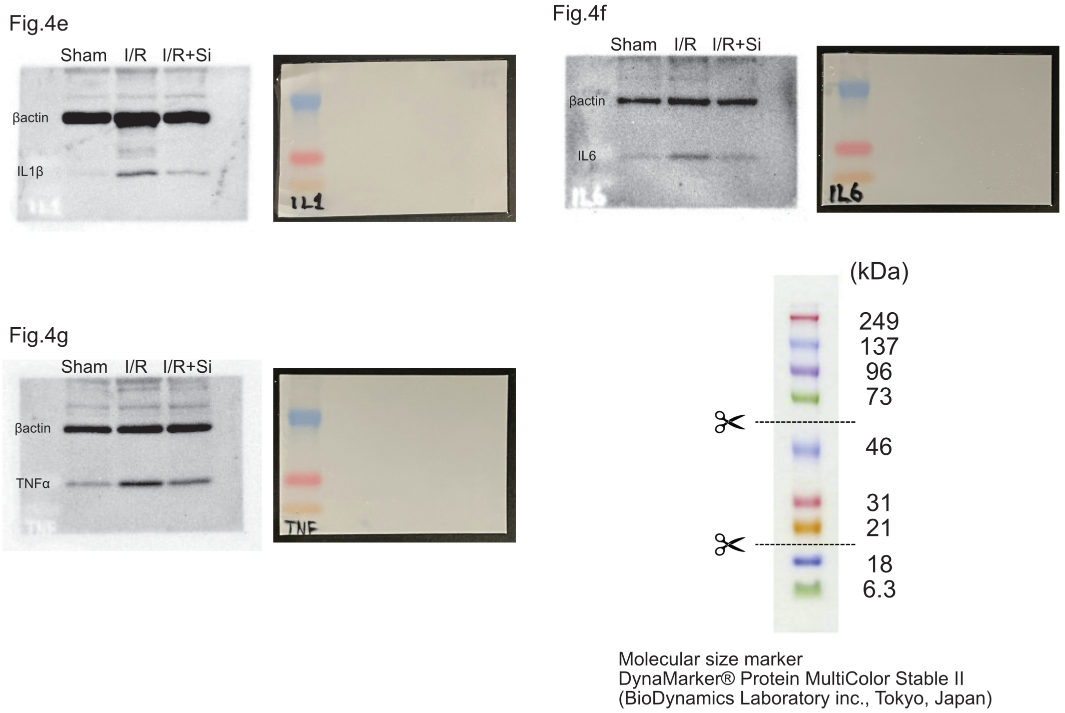

Supplementary Figure 1.

Original images of Fig.4e-f. Representative samples of each group were used. These membranes were cut around the predicted molecular weight prior to hybridization with antibodies.

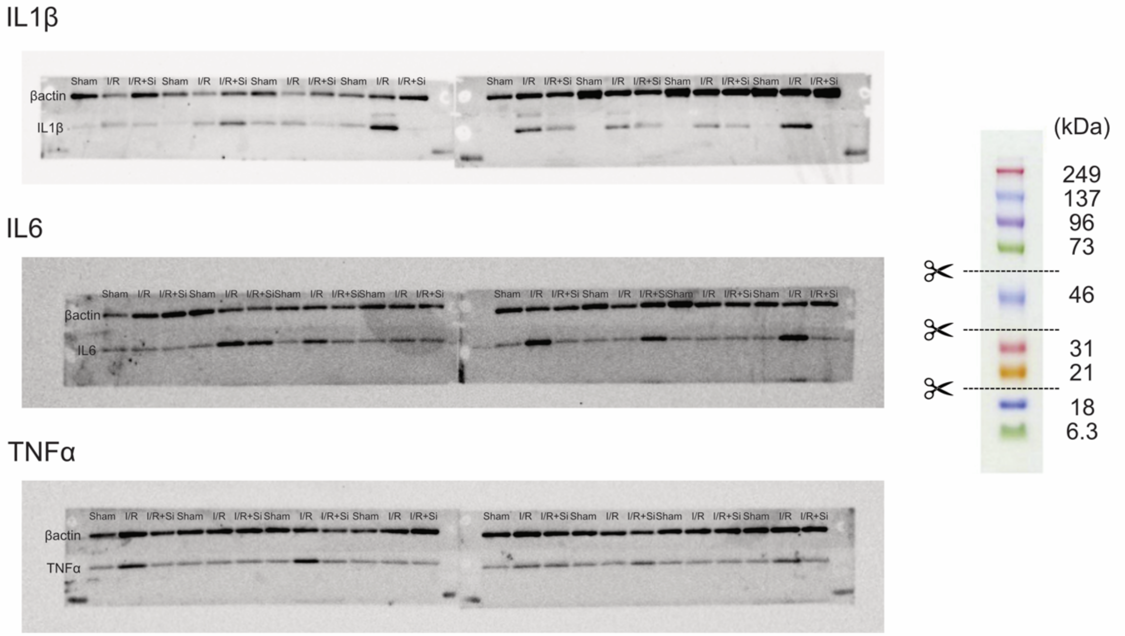

Supplementary Figure 2.

All biological replicates of Western blotting. These membranes were cut around each predicted molecular weight prior to hybridization with antibodies. All samples were derived from the same experiment and gels/blots were processed in parallel.
